# Supplementary material for: Machine Learning Accelerates Crystallization for Structure Determination
Source: Angew Chem Int Ed Engl. 2026 May 4;65(25):e1218503. doi: 10.1002/anie.1218503 (PMC13266958; doi:10.1002/anie.1218503)

checkCIF/PLATON report

Structure factors have been supplied for datablock(s) lcz067

THIS REPORT IS FOR GUIDANCE ONLY. IF USED AS PART OF A REVIEW PROCEDURE FOR PUBLICATION, IT SHOULD NOT REPLACE THE EXPERTISE OF AN EXPERIENCED CRYSTALLOGRAPHIC REFEREE.

No syntax errors found. CIF dictionary Interpreting this report

Datablock: lcz067

Bond precision:	C-C = 0.0163 A		Wavelength=1.54184
Cell:	a=12.9257(2)	b=13.2495(2)	c=29.9273(5)
	alpha=79.982(1)	beta=79.499(1)	gamma=60.911(2)
Temperature:	100 K		

	Calculated	Reported
Volume	4382.05(15)	4382.05(14)
Space group	P -1	P -1
Hall group	-P 1	-P 1
Moiety formula	C15 H3 Ag3 F18 N6, C18 H14 O4 [+ solvent]	C15 H3 Ag3 F18 N6, C18 H14 O4
Sum formula	C33 H17 Ag3 F18 N6 O4 [+ solvent]	C33 H17 Ag3 F18 N6 O4
Mr	1227.14	1227.14
Dx,g cm-3	1.860	1.860
Z	4	4
Mu (mm-1)	11.757	11.757
F000	2368.0	2368.0
F000’	2379.11	
h,k,lmax	16,16,37	15,16,36
Nref	17660	16439
Tmin,Tmax	0.136,0.152	0.677,1.000
Tmin’	0.045	

Correction method= # Reported T Limits: Tmin=0.677 Tmax=1.000
AbsCorr = MULTI-SCAN

Data completeness= 0.931   Theta(max)= 73.554

R(reflections)= 0.0559(13945)   wR2(reflections)= 0.1450(16439)

S = 1.051   Npar= 1155

The following ALERTS were generated. Each ALERT has the format
test-name_ALERT_alert-type_alert-level.
Click on the hyperlinks for more details of the test.

Alert level C

```
PLAT029_ALERT_3_C _diffrn_measured_fraction_theta_full value Low .      0.968 Why?
PLAT094_ALERT_2_C Ratio of Maximum / Minimum Residual Density ....       2.05 Report
PLAT213_ALERT_2_C Atom F02X            has ADP max/min Ratio .....        3.6 prolat
PLAT213_ALERT_2_C Atom F036            has ADP max/min Ratio .....        4.0 prolat
PLAT213_ALERT_2_C Atom F02G            has ADP max/min Ratio .....        3.3 prolat
PLAT213_ALERT_2_C Atom F02N            has ADP max/min Ratio .....        3.8 prolat
PLAT220_ALERT_2_C NonSolvent   Resd 1  F   Ueq(max)/Ueq(min) Range        4.5 Ratio
PLAT234_ALERT_4_C Large Hirshfeld Difference O02K     --C034     .       0.16 Ang.
PLAT250_ALERT_2_C Large U3/U1 Ratio for <U(i,j)> Tensor(Resd    1)        2.1 Note
PLAT250_ALERT_2_C Large U3/U1 Ratio for <U(i,j)> Tensor(Resd    2)        2.3 Note
PLAT250_ALERT_2_C Large U3/U1 Ratio for <U(i,j)> Tensor(Resd    4)        2.2 Note
PLAT309_ALERT_2_C Single Bonded Oxygen (C-O > 1.3 Ang) ..........        O00L Check
PLAT309_ALERT_2_C Single Bonded Oxygen (C-O > 1.3 Ang) ..........        O00R Check
PLAT309_ALERT_2_C Single Bonded Oxygen (C-O > 1.3 Ang) ..........        O035 Check
PLAT342_ALERT_3_C Low Bond Precision on  C-C Bonds ..............    0.01633 Ang.
PLAT362_ALERT_2_C Short  C(sp3)-C(sp2) Bond  C01J     - C021     .       1.40 Ang.
PLAT362_ALERT_2_C Short  C(sp3)-C(sp2) Bond  C2       - C03E     .       1.39 Ang.
PLAT906_ALERT_3_C Large K Value in the Analysis of Variance ......      2.181 Check
PLAT911_ALERT_3_C Missing FCF Refl Between Thmin & STh/L=    0.600        507 Report
               -5-15  5,   3 15  9, -12  1 10,  11  3 13,   4 15 13,   3 -6 21,
                2 -9 22,   3 -8 22,   5 -7 22,   2 -6 22,   5 -4 22,   7 -2 22,
                0 -9 23,   1 -9 23,   2 -8 23,   3 -8 23,   3 -7 23,   4 -7 23,
                1 -6 23,   2 -6 23,   5 -6 23,   6 -6 23,   3 -5 23,   7 -5 23,
               -1 -9 24,   0 -9 24,   0 -8 24,   1 -8 24,   2 -8 24,   1 -7 24,
                   (  477 More Missing: see the .ckf listing file)
PLAT934_ALERT_3_C Number of (Iobs-Icalc)/Sigma(W) > 10 Outliers ..          1 Check
                5 13  4,
PLAT971_ALERT_2_C Check Calcd Resid. Dens.  0.86Ang From N011            2.20 eA-3
PLAT971_ALERT_2_C Check Calcd Resid. Dens.  1.62Ang From Ag2             2.20 eA-3
PLAT971_ALERT_2_C Check Calcd Resid. Dens.  1.29Ang From F036            2.10 eA-3
PLAT971_ALERT_2_C Check Calcd Resid. Dens.  1.38Ang From N013            2.09 eA-3
PLAT971_ALERT_2_C Check Calcd Resid. Dens.  0.92Ang From N01A            1.89 eA-3
PLAT971_ALERT_2_C Check Calcd Resid. Dens.  1.61Ang From Ag4             1.71 eA-3
PLAT971_ALERT_2_C Check Calcd Resid. Dens.  1.36Ang From F02X            1.69 eA-3
PLAT971_ALERT_2_C Check Calcd Resid. Dens.  1.40Ang From N01C            1.68 eA-3
PLAT977_ALERT_2_C Check Negative Difference Density on H01A      .      -0.38 eA-3
PLAT977_ALERT_2_C Check Negative Difference Density on H01B      .      -0.37 eA-3
PLAT977_ALERT_2_C Check Negative Difference Density on H03A      .      -0.37 eA-3
PLAT977_ALERT_2_C Check Negative Difference Density on H03C      .      -0.39 eA-3
```

```
PLAT977_ALERT_2_C Check Negative Difference Density on H34       .       -0.43 eA-3
```

Alert level G

```
PLAT003_ALERT_2_G Number of Uiso or U(i,j)  Restrained non-H-Atoms         20 Report
PLAT083_ALERT_2_G SHELXL Second Parameter in WGHT  Unusually Large      42.22 Why ?
PLAT177_ALERT_4_G The CIF-Embedded .res File Contains DELU Records          2 Report
PLAT178_ALERT_4_G The CIF-Embedded .res File Contains SIMU Records          1 Report
PLAT186_ALERT_4_G The CIF-Embedded .res File Contains ISOR Records          2 Report
PLAT188_ALERT_3_G A Non-default SIMU Restraint Value has been used     0.0100 Report
PLAT192_ALERT_3_G A Non-default DELU Restraint Value for SecondPar     0.0200 Report
PLAT192_ALERT_3_G A Non-default DELU Restraint Value for SecondPar     0.0200 Report
PLAT242_ALERT_2_G Low    'MainMol' Ueq as Compared to Neighbors of       C01W Check
PLAT242_ALERT_2_G Low    'MainMol' Ueq as Compared to Neighbors of       C01X Check
PLAT242_ALERT_2_G Low    'MainMol' Ueq as Compared to Neighbors of       C02B Check
PLAT242_ALERT_2_G Low    'MainMol' Ueq as Compared to Neighbors of       C02O Check
PLAT242_ALERT_2_G Low    'MainMol' Ueq as Compared to Neighbors of       C039 Check
PLAT242_ALERT_2_G Low    'MainMol' Ueq as Compared to Neighbors of       C03G Check
PLAT242_ALERT_2_G Low    'MainMol' Ueq as Compared to Neighbors of       C025 Check
PLAT242_ALERT_2_G Low    'MainMol' Ueq as Compared to Neighbors of       C02F Check
PLAT242_ALERT_2_G Low    'MainMol' Ueq as Compared to Neighbors of       C02M Check
PLAT242_ALERT_2_G Low    'MainMol' Ueq as Compared to Neighbors of       C02R Check
PLAT242_ALERT_2_G Low    'MainMol' Ueq as Compared to Neighbors of       C02U Check
PLAT242_ALERT_2_G Low    'MainMol' Ueq as Compared to Neighbors of       C02W Check
PLAT398_ALERT_2_G Deviating  C-O-C   Angle From 120 for O00S     .      104.6 Degree
PLAT398_ALERT_2_G Deviating  C-O-C   Angle From 120 for O01U     .      107.5 Degree
PLAT434_ALERT_2_G Short Inter HL..HL Contact F00B     ..F01R     .       2.76 Ang.
                                                  x,1+y,z  =     1_565 Check
PLAT434_ALERT_2_G Short Inter HL..HL Contact F00V     ..F036     .       2.80 Ang.
                                                  x,1+y,z  =     1_565 Check
PLAT606_ALERT_4_G Solvent Accessible VOID(S) in  Crystal Structure          ! Info
PLAT720_ALERT_4_G Number of Unusual/Non-Standard Labels ..........        151 Note
              F00A    F00B    F00C    F00D    F00E    F00F    F00G    F00H
              F00I    F00J    F00K    O00L    O00M    F00N    F00O    F00P
              N00Q    O00R    O00S    F00T    F00U    F00V    F00W    F00X
              F00Y    F00Z    F010    N011    N012    N013    N014    N015
              N016    N017    F018    F019    N01A    N01B    N01C    F01D
              F01E    F01F    F01G    N01H    F01I    C01J    H01A    H01B
              C01K    C01L    H01L    C01M    C01O    C01P    H01P    C01Q
              F01R    C01S    F01T    O01U    C01V    C01W    C01X    C01Y
              C01Z    C020    C021    C022    H022    C023    C024    C025
              C026    H026    C027    H027    C028    H028    F029    C02A
              H02A    C02B    C02C    C02D    C02E    H02E    C02F    F02G
              C02H    H02H    C02I    O02J    O02K    C02L    H02L    C02M
              F02N    C02O    C02P    C02Q    H02Q    C02R    C02S    C02T
              C02U    C02V    H02V    C02W    F02X    C02Y    H02B    H02C
              H02D    C02Z    H02Z    C030    H030    C031    C032    H032
              C033    C034    O035    F036    C037    C038    H038    C039
              C03A    C03B    H03B    C03E    H03A    H03C    C03F    C03G
              C03I    H03I    C03J    H03J    C03K    C03L    H03L    C03O
              H03D    H03E    H03F    C03P    H03P    C03Q    H03Q
PLAT860_ALERT_3_G Number of Least-Squares Restraints .............        128 Note
PLAT868_ALERT_4_G ALERTS Due to the Use of _smtbx_masks Suppressed          ! Info
PLAT883_ALERT_1_G Absent Datum for _atom_sites_solution_primary ..     Please Do !
PLAT910_ALERT_3_G Missing FCF Reflection(s) Below Theta(Min)[Deg]=       3.84 Note
                0  0  1,   0  0  2,
PLAT912_ALERT_4_G Missing # of FCF Reflections Above STh/L=  0.600        687 Note
```

```
PLAT969_ALERT_5_G The 'Henn et al.' R-Factor-gap value ..........      4.117 Note
              Predicted wR2: Based on SigI**2  3.52 or SHELX Weight 13.80
PLAT978_ALERT_2_G Number C-C Bonds with Positive Residual Density.         0 Info
```

```
   0 ALERT level A = Most likely a serious problem - resolve or explain
   0 ALERT level B = A potentially serious problem, consider carefully
  33 ALERT level C = Check. Ensure it is not caused by an omission or oversight
  33 ALERT level G = General information/check it is not something unexpected

   1 ALERT type 1 CIF construction/syntax error, inconsistent or missing data
  46 ALERT type 2 Indicator that the structure model may be wrong or deficient
  10 ALERT type 3 Indicator that the structure quality may be low
   8 ALERT type 4 Improvement, methodology, query or suggestion
   1 ALERT type 5 Informative message, check
```

Validation response form

Please find below a validation response form (VRF) that can be filled in and pasted into your CIF.

```
# start Validation Reply Form
_vrf_PLAT029_lcz067
;
PROBLEM: _diffrn_measured_fraction_theta_full value Low .      0.968 Why?
RESPONSE: ...
;
_vrf_PLAT094_lcz067
;
PROBLEM: Ratio of Maximum / Minimum Residual Density ....       2.05 Report
RESPONSE: ...
;
_vrf_PLAT213_lcz067
;
PROBLEM: Atom F02X            has ADP max/min Ratio .....        3.6 prolat
RESPONSE: ...
;
_vrf_PLAT220_lcz067
;
PROBLEM: NonSolvent   Resd 1  F   Ueq(max)/Ueq(min) Range        4.5 Ratio
RESPONSE: ...
;
_vrf_PLAT234_lcz067
;
PROBLEM: Large Hirshfeld Difference O02K     --C034     .       0.16 Ang.
RESPONSE: ...
;
_vrf_PLAT250_lcz067
;
PROBLEM: Large U3/U1 Ratio for <U(i,j)> Tensor(Resd    1)        2.1 Note
RESPONSE: ...
;
_vrf_PLAT309_lcz067
;
PROBLEM: Single Bonded Oxygen (C-O > 1.3 Ang) ..........       O00L Check
RESPONSE: ...
;
```

```
_vrf_PLAT342_lcz067
;
PROBLEM: Low Bond Precision on  C-C Bonds ...............    0.01633 Ang.
RESPONSE: ...
;
_vrf_PLAT362_lcz067
;
PROBLEM: Short  C(sp3)-C(sp2) Bond  C01J     - C021     .       1.40 Ang.
RESPONSE: ...
;
_vrf_PLAT906_lcz067
;
PROBLEM: Large K Value in the Analysis of Variance ......      2.181 Check
RESPONSE: ...
;
_vrf_PLAT911_lcz067
;
PROBLEM: Missing FCF Refl Between Thmin & STh/L=    0.600        507 Report
RESPONSE: ...
;
_vrf_PLAT934_lcz067
;
PROBLEM: Number of (Iobs-Icalc)/Sigma(W) > 10 Outliers ..          1 Check
RESPONSE: ...
;
_vrf_PLAT971_lcz067
;
PROBLEM: Check Calcd Resid. Dens.  0.86Ang From N011            2.20 eA-3
RESPONSE: ...
;
_vrf_PLAT977_lcz067
;
PROBLEM: Check Negative Difference Density on H01A      .      -0.38 eA-3
RESPONSE: ...
;
# end Validation Reply Form
```

It is advisable to attempt to resolve as many as possible of the alerts in all categories. Often the minor alerts point to easily fixed oversights, errors and omissions in your CIF or refinement strategy, so attention to these fine details can be worthwhile. It is up to the individual to critically assess their own results and, if necessary, seek expert advice.

PLATON version of 17/09/2025; check.def file version of 17/09/2025

---

duplicate check

No duplication found

Datablock lcz067 - ellipsoid plot

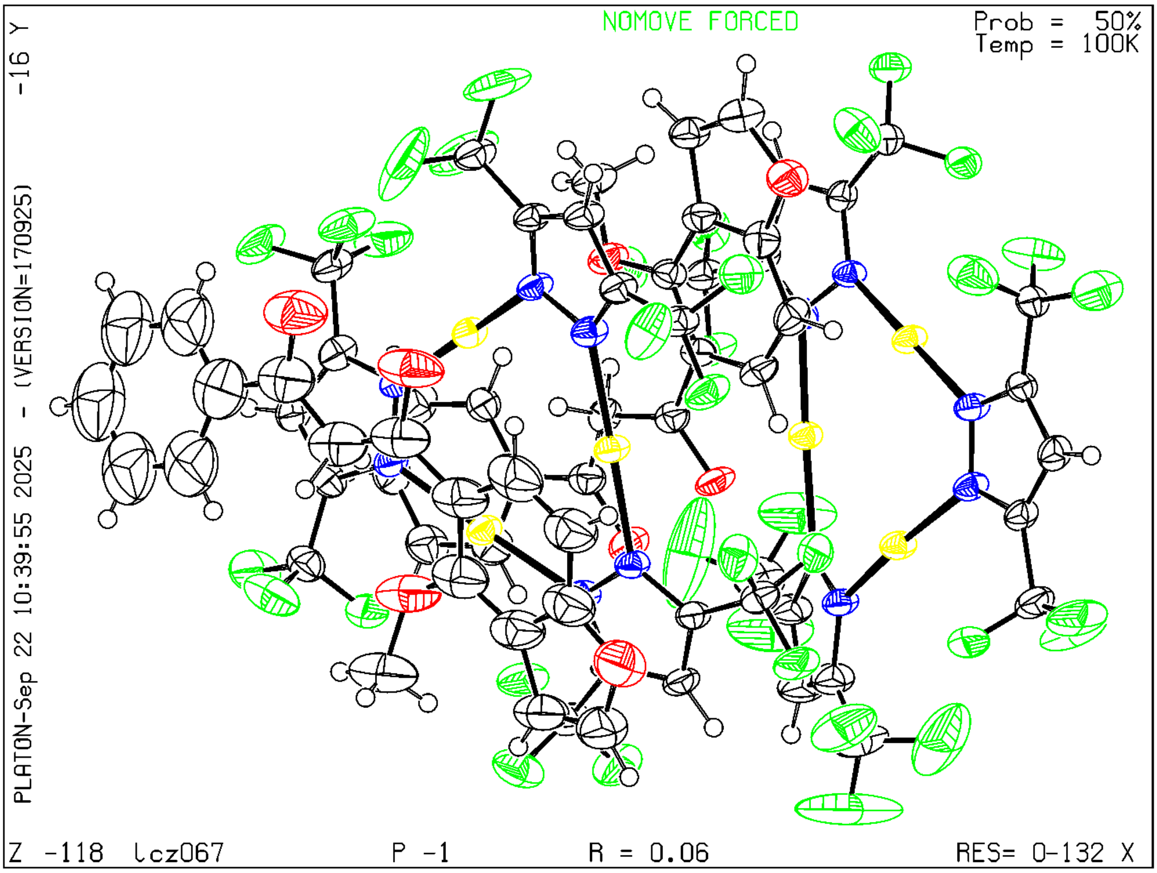

Supplement: Supplementary file 2 — Supporting File 2: anie72462‐sup‐0002‐DataFile.zip. [file ANIE-65-e1218503-s002.zip › supplementary_materials_crystallographic_data submit.zip!/cif_and_checkcif(1-114)/Ag3Pz3_67/Ag3Pz3_67_checkcif.pdf]
